# Euglycemic Diabetic Ketoacidosis after a Single Dose of Empagliflozin in a Patient with Pancreatitis

**DOI:** 10.3390/clinpract11020031

**Published:** 2021-04-06

**Authors:** Marta Brandão Calçada, Luís Fernandes, Rita Soares Costa, Sara Montezinho, Filipa Martins Duarte, Luísa Frutuoso, Ana Raquel Freitas

**Affiliations:** 1 Internal Medicine Department, Centro Hospitalar de Entre o Douro e Vouga, 4520-211 Santa Maria da Feira, Portugal; luisp.fernandes@outlook.com (L.F.); ritafscosta@gmail.com (R.S.C.); saramontezinho@gmail.com (S.M.); filipamartinsduarte@hotmail.com (F.M.D.); rakel.freitas@hotmail.com (A.R.F.); 2 Surgery Department, Centro Hospitalar de Entre o Douro e Vouga, 4520-211 Santa Maria da Feira, Portugal; luisafrutuoso04@gmail.com

**Keywords:** diabetic ketoacidosis, sodium-glucose cotransporter 2 inhibitors, diabetes, empagliflozin

## Abstract

Sodium-glucose cotransporter 2 inhibitors (SGLT2i) are the most recently approved drug class for the treatment of type 2 diabetes mellitus (T2D). Although they are largely well-tolerated, their intake has been associated with euglycemic diabetic ketoacidosis (DKA) in some rare cases. We report the case of a 70-year-old male with type 2 diabetes and no history of DKA, who started therapy with empagliflozin one day before presenting with acute pancreatitis and laboratory findings consistent with euglycemic DKA. SGLT2i can induce euglycemic DKA from the first dose. Given the atypical presentation, a high degree of clinical suspicion is required to recognize this complication.

## 1. Introduction

Sodium-glucose cotransporter 2 inhibitors (SGLT2i) are the most recently approved drug class for type 2 diabetes mellitus treatment (T2D). This group of oral glucose-lowering drugs acts on the kidney by inhibiting glucose reabsorption to the blood in the proximal tubule, consequently leading to glucosuria [1].

On 1st August 2014, empagliflozin, a SGLT2i, was approved by the US Food and Drug Administration (FDA) as an adjunctive therapy to diet and exercise to improve glycemic control in adults with TD2 [2]. On 2nd December, 2016, a new indication for empagliflozin was FDA approved: to reduce the risk of cardiovascular death in adult patients with T2D and cardiovascular disease [3].

Although this drug class is generally well-tolerated, in May 2015 the FDA emitted a warning on the potential risk of diabetic ketoacidosis (DKA) with unusually low-to-moderately elevated levels of serum glucose (Euglycemic DKA), following the use of SGLT2i [4]. The proposed mechanisms of Euglycemic DKA induced by SGLT2i are as follows: by reducing blood glucose levels, SGLT2i decrease the secretion of endogenous insulin by pancreatic b-cells. This in turn stimulates pancreatic a-cells, leading to increased glucagon secretion. SGLT2i also directly stimulate a-cells, thereby increasing plasma glucagon concentration and also promoting hepatic ketogenesis [5] In addition, SGLT2i increase the reabsorption of acetoacetate in the renal tubules, which raises ketonemia [5].

The rare presentation of Euglycemic DKA may lead to a delay in diagnosis and treatment with serious and potentially fatal consequences.

In this article, we describe an uncommon presentation of empagliflozin-associated euglycemic DKA in a patient with T2D, only one day after empagliflozin introduction. 

## 2. Case Report

Seventy-year-old man with a past medical history of alcoholism, overweight (Body Mass Index of 28.9 kg/m^2^) and T2D for more than 10 years, well-controlled with a combination of dipeptidyl peptidase-4 inhibitor, biguanide and sulfonylurea (last known HbA1c 7.8%; average blood glucose levels of 162 mg/dL), with no previous episodes of DKA. 

He presented to the emergency department with epigastric pain and vomiting for 3 days. The day before, he had been prescribed empagliflozin for hyperglycemia. At admission, he was alert and oriented, with dehydrated skin and mucous membranes. The blood pressure was 136/90 mmHg with a pulse of 116 bpm. He was febrile (tympanic temperature −38 °C) and had tachypnea. Cardiac and pulmonary auscultation was clear. There was diffuse abdominal tenderness but with no signs of peritoneal irritation. Urinary output was >0.5 mL/kg/h.

Labs showed normal renal function and no electrolyte abnormalities. Amylase (253 U/L) and lipase (493 U/L) were elevated. There was also hyperglycemia (188 mg/dL), ketonemia (6.6 mmol/L) and metabolic acidemia with an increased anion gap (AG)—pH 7.28; pCO_2_ 22 mmHg; HCO_3_^−^ 10.3 mEq/L; lactate 2.0 mmol/L; AG 26 mmol/L—which worsened despite aggressive intravenous fluid replacement. Other causes of metabolic acidosis with an increased AG were excluded (renal insufficiency lactic acidosis and intoxication either by methanol, ethylene glycol or salicylates) and the diagnosis of SGLT2 Inhibitor-Associated Euglycemic DKA was assumed, aggravated in the context of an acute pancreatitis and decreased carbohydrate intake. Normal saline with dextrose and intravenous continuous insulin perfusion were started, alongside vital signs, hourly blood glucose, serum ketones and potassium were monitored. Afterwards, he was admitted to the surgical ward, with daily Internal Medicine evaluation, under a basal-bolus insulin regimen, with a favorable clinical evolution. After hospital discharge, the patient remained on basal-bolus insulin therapy, and was also referred to a specialized diabetes mellitus consultation for follow-up.

## 3. Discussion

DKA is a serious and potentially fatal T2D complication, occurring because of a profound insulin deficit. Euglycemic DKA is an unusual subset, characterized by metabolic acidosis with a pH < 7.3 and serum bicarbonate <18 mEq/L, ketosis, and serum glucose >200 mg/dL [6,7].

DKA incidence in T2D is 1.34/million people per year [8]. In opposition, the incidence rate of euglycemic DKA is uncertain. Insulin dose reduction or cessation, severe acute illness, dehydration, surgery, low-carbohydrate diet, or excessive alcohol intake are among the risk factors [9,10].

In the present case, acute pancreatitis was considered the main trigger for DKA development. Acute pancreatitis is associated with decreased oral intake, possibly leading to a catabolic state with subsequent ketone bodies formation in the context of SGLT2i use. Excessive alcohol consumption may have also favored DKA appearance. However, it is unexpected that a single drug dose administration could have caused such a significant impact, since the average reported time from SGLT2i start or dose increasement to euglycemic DKA onset is 43 days (range 1 to 365 days) [4].

Furthermore, the development of acute pancreatitis was related to incretin-based therapeutics in varied randomized controlled trials [11]. In our patient, dipeptidyl peptidase-4 inhibitor features as a potential cause of acute pancreatitis; however, there is no established causality [12]. Nonetheless, drug discontinuation is recommended after an episode of acute pancreatitis [12], which was done.

Treatment for SGLT2i-associated euglycemic DKA is identical to conventional DKA, besides immediate drug withdrawal. Fortunately, in our case, the identification of the predisposing factors allowed for a rapid recognition of this entity and led to a timely treatment.

## 4. Conclusions

Although euglycemic DKA is a known SGLT2i adverse effect, its rarity and atypical presentation requires a high degree of clinical suspicion. Furthermore, as our case highlights, this diagnosis should be kept in mind as a possible complication since SGLT2i first administration. This is a potentially fatal condition in which early diagnosis and treatment allow for a complete clinical recovery.

## Data Availability

No new data were created or analyzed in this study. Data sharing is not applicable to this article.
